# A systematic review of inequalities in the uptake of, adherence to, and effectiveness of behavioral weight management interventions in adults

**DOI:** 10.1111/obr.13438

**Published:** 2022-03-03

**Authors:** Jack M. Birch, Rebecca A. Jones, Julia Mueller, Matthew D. McDonald, Rebecca Richards, Michael P. Kelly, Simon J. Griffin, Amy L. Ahern

**Affiliations:** ^1^ MRC Epidemiology Unit University of Cambridge Cambridge UK; ^2^ Curtin School of Population Health Curtin University Perth Western Australia Australia; ^3^ Department of Public Health and Primary Care University of Cambridge Cambridge UK

**Keywords:** inequalities, interventions, obesity, weight management

## Abstract

The extent to which behavioral weight management interventions affect health inequalities is uncertain, as is whether trials of these interventions directly consider inequalities. We conducted a systematic review, synthesizing evidence on how different aspects of inequality impact uptake, adherence, and effectiveness in trials of behavioral weight management interventions. We included (cluster‐) randomized controlled trials of primary care‐applicable behavioral weight management interventions in adults with overweight or obesity published prior to March 2020. Data about trial uptake, intervention adherence, attrition, and weight change by PROGRESS‐Plus criteria (place of residence, race/ethnicity, occupation, gender, religion, education, socioeconomic status, social capital, plus other discriminating factors) were extracted. Data were synthesized narratively and summarized in harvest plots. We identified 91 behavioral weight loss interventions and 12 behavioral weight loss maintenance interventions. Fifty‐six of the 103 trials considered inequalities in relation to at least one of intervention or trial uptake (*n* = 15), intervention adherence (*n* = 15), trial attrition (*n* = 32), or weight outcome (*n* = 34). Most trials found no inequalities gradient. If a gradient was observed for trial uptake, intervention adherence, and trial attrition, those considered “more advantaged” did best. Alternative methods of data synthesis that enable data to be pooled and increase statistical power may enhance understanding of inequalities in behavioral weight management interventions.

**Abbreviations**
BMI: body mass index
OECD: Organization for Economic Collaboration and Development
PRISMA: Preferred Reporting Items for Systematic Reviews and Meta‐Analysis
RCT: randomized controlled trial
SES: socioeconomic status
USPSTF: United States Preventive Services Taskforce
WLMs: behavioral weight loss maintenance interventions
WLs: behavioral weight loss interventions

## 1 INTRODUCTION

Overweight and obesity are associated with an increased risk of several non‐communicable diseases including type 2 diabetes, cardiovascular disease, and some cancers (e.g., bowel and post‐menopausal breast).^1^, ^2^ Behavioral weight management interventions have been shown to be effective in promoting (behavioral weight loss interventions [WLs]) and maintaining (behavioral weight loss maintenance interventions [WLMs]) weight loss in those with overweight or obesity.^3^, ^4^ Interventions designed to support individuals to change their health behaviors, such as behavioral weight management interventions, typically require a high amount of personal agency (such as time, resource and education) in order to be effective.^5^, ^6^ In this way, they may benefit advantaged groups with higher personal agency more than those less advantaged. Consequently, behavioral weight management interventions may be inequitable and exacerbate health inequalities.^5^, ^6^ Health inequalities are systemic and avoidable differences in health outcomes between difference groups in a population.^7^ Inequalities may arise at various stages of an intervention, such as uptake, adherence, or outcome and can occur across characteristics summarized by the PROGRESS‐Plus framework (place of residence, race/ethnicity, occupation, gender/sex, education, socioeconomic status [SES], social capital, plus other factors for which discrimination could occur such as age and sexual orientation).^8^

Several previous systematic reviews have considered the relationship between characteristics where inequalities may occur and interventions for overweight or obesity.^9^, ^10^, ^11^, ^12^, ^13^, ^14^ The United States Preventive Services Taskforce (USPSTF) considered the overall effectiveness of behavioral and pharmacological interventions for overweight and obesity^3^ and provided narrative comment about some aspects of inequality. The authors found that unless the intervention was targeted towards a specific ethnicity, ethnicity and SES were frequently not reported. Where these were reported, most participants were White and of mid‐to‐high SES.

The other systematic reviews we identified focused on a single characteristic where inequality might occur (one of race or ethnicity, gender, and SES). Seven reviews focused on race or ethnicity.^9^, ^10^, ^11^, ^12^, ^13^, ^14^, ^15^ Two only included interventions that were targeted towards Latinos in America,^9^, ^14^ and one only included interventions that were tailored towards African American women.^10^ Four systematic reviews included studies if they reported more than one race or ethnicity represented in their sample. Haughton et al.^13^ found that only 2/60 WL studies conducted analysis of differential attendance by ethnicity and 8/60 conducted analysis of differential outcome by ethnicity. Across 71 trials of interventions that focused on using technology for weight loss, Rosenbaum et al. found there was low enrolment (trial uptake) of racial minorities.^12^ Fitzgibbon et al. included all trials (*n* = 25) that reported including Black women (not only trials of interventions that were targeted towards Black women).^11^ They found that Black women had lower weight loss and higher study attrition than other groups but no differences in intervention adherence. Tussing‐Humphreys et al. reviewed 17 studies of WLMs that included African American women^15^ and found that African American women generally lost less weight during the weight loss phase and regained more weight during the maintenance phase when compared with Caucasian women.

We also identified systematic reviews that considered gender in behavioral weight management trials.^16^, ^17^, ^18^ These reviews found that males are generally underrepresented in trials of WLs and WLMs^16^ that male‐only interventions may effectively engage and assist men in achieving weight loss but,^17^ in interventions that are delivered to males and females, achieved weight loss was similar.^18^

Two systematic reviews synthesized data on inequalities by SES. Olstad et al., in a review of universal policies for obesity in adults and children, found no evidence to support the theory that interventions targeted towards individuals/households, such as behavioral weight management interventions, were more likely to be inequitable than population‐level interventions.^19^ However, Olstad et al. noted that this may have been due to the few “agentic” (i.e., interventions requiring a high amount of personal agency in order to take effect) policies included in the review. In an earlier review, Hillier‐Brown et al. considered effectiveness of individual‐, community‐, and population‐level interventions at reducing socioeconomic inequalities in obesity.^20^ The authors identified evidence from interventions targeting deprived groups, rather than those aimed at the population more generally. The authors only included studies reporting differential effects by SES and only looked at outcome measures rather than process measures (such as uptake and trial attrition).

The highlighted systematic reviews generally focused on a single PROGRESS‐Plus characteristic. It is useful to examine all of the PROGRESS‐Plus characteristics in a single review to gain a broader understanding of inequalities and identify any under‐researched characteristics. Furthermore, some of the previous systematic reviews restricted their inclusion criteria to specific races or ethnicities^9^, ^14^ or a specific subcategory of behavioral intervention.^12^ Few reviews assessed if there were inequalities at trial stages other than weight outcome, especially in reviews that focused on SES.^19^, ^20^ It is important to understand at what stage inequalities occur in order to effectively address them.

Therefore, we aimed to:

1. Summarize the number and characteristics of trials of behavioral weight management interventions that report measures of inequalities as defined by the PROGRESS‐Plus criteria.
2. Identify, describe, and synthesize trial data on inequalities in the uptake of, adherence to, and effectiveness of behavioral weight management interventions.
3. Synthesize data on differential attrition in trials of behavioral weight management interventions.

## 2 METHODS

This review was completed according to the Preferred Reporting Items for Systematic Reviews and Meta‐Analysis (PRISMA) guidelines and the PRISMA‐Equity extension.^21^, ^22^ Full details of the methods were published in the protocol,^23^ and the review was registered on PROSPERO (CRD42020173242).

### 2.1 Eligibility criteria

1. Participants: adults aged 18 years and over with overweight or obesity (body mass index [BMI] ≥ 25 kg/m^2^ with no upper limit) who were deemed suitable (either by the applicable study team or healthcare practitioner) for WLs or WLMs. Participants may have additional risk factors such as hypertension, dyslipidemia, impaired glucose tolerance, or impaired fasting glucose. Studies were excluded if the population was not selected based on a weight‐related measure, included participants with BMI < 25 kg/m^2^ were selected based on having a chronic disease where weight loss is part of disease management or being pregnant, or if the intervention was targeted at parents to change behavior of children. Only studies conducted in member countries of the Organization for Economic Collaboration and Development (OECD) were eligible.
2. Interventions: behavioral weight management interventions with the primary aim of supporting weight loss or weight loss maintenance. Studies were included if they were conducted in, or were applicable to, primary care settings. Interventions may have been delivered alone or as part of a wider multicomponent intervention targeting diet and nutrition, physical activity, sedentary behavior, or a combination of these. Interventions may include but were not limited to, assessment with feedback, advice, provider training, goal‐setting, or exercise referral. Studies of pharmacological and surgical interventions were excluded unless the trial included behavioral only and control arms. Interventions were considered feasible for application to primary care if they were conducted in a healthcare setting or are widely available in the community at a national or regional level (such as commercial weight loss programs, text‐message based interventions); examples of settings that are not relevant to primary care include interventions delivered in inpatient settings or in residential care homes.
3. Comparators: wait‐list control, usual care, or minimal intervention (such as generic print or electronic materials).
4. Outcomes: studies must report a weight outcome (weight change in kg, ≥5% weight loss, or change in waist circumference) at either the 12‐ or 18‐month follow‐up.
5. Study designs: randomized or cluster‐randomized controlled trials (RCTs). Studies that were not available in English were excluded.

### 2.2 Search strategy and information sources

We adopted a two‐stage search strategy to identify relevant publications. The first stage involved identifying “parent” RCTs^24^ (trials included in the USPSTF review^3^). We then replicated the search used in the USPSTF review to identify relevant trials published since the USPSTF database search date (June 2017). The updated search was performed on March 5, 2020 in Medline, Cochrane CENTRAL, and PsycInfo (search strategy available in Supplementary File 1a). We used the USPSTF review due to the comprehensive search strategy used in the review that increased the likelihood of identifying relevant trails of behavioral weight management interventions for inclusion.

The second stage involved conducting a further Medline search to identify all further publications from each parent RCT.

### 2.3 Study selection

All behavioral weight management interventions included in the USPSTF report were included in the review. The titles and abstracts from the updated search were independently screened by two investigators (JMB and RAJ) using Covidence systematic review software (Veritas Health Innovation, Melbourne, Australia). Any discrepancies were resolved by consensus. Full texts of studies identified from the title and abstract screening as being potentially relevant were screened independently by two investigators (JMB and RAJ), and conflicts resolved through discussion. Trials already identified from the USPSTF report and included in our search results were de‐duplicated at this stage. Where necessary, conflicts were discussed with a third investigator (ALA) to reach consensus.

### 2.4 Data extraction

Data extraction was completed by one investigator (JMB) and checked by a second investigator (RAJ, JM, MDMcD, and RR). The data extraction form was developed using the Cochrane Public Health Group data extraction form, the Consolidated Standards of Reporting Trials 2010 statement, the Template for Intervention Description and Replication checklist, and the PROGRESS‐Plus criteria.^8^, ^25^, ^26^, ^27^

### 2.5 Outcomes

#### 2.5.1 Trial and intervention uptake

Trial uptake was defined as participants accepting an invitation to participate in a trial of a behavioral weight management intervention. Therefore, we defined differential trial uptake as whether those who accepted invitation to participate differed from those who declined to take part by a measure of a PROGRESS‐Plus criterion.

Intervention uptake was defined as a participant attending or completing at least one session of the intervention. We defined differential intervention uptake as a statistically significant difference between participants attending at least one session of an intervention versus those who did not, by a measure of a PROGRESS‐Plus characteristic.

#### 2.5.2 Intervention adherence

Adherence was defined as number of intervention sessions attended out of those offered or as engagement with any intervention component (such as completion of food diaries or number of times logged into a mobile application). Differential adherence was defined as a statistically significant difference in the number of sessions of an intervention attended or engagement with an intervention component by a measure of a PROGRESS‐Plus characteristic.

#### 2.5.3 Trial attrition

Trial attrition was defined as those lost to follow‐up at the 12‐month follow‐up. Where attrition for the 12‐month time point was not reported, data reported up until 18 months of follow‐up were extracted instead. We considered differential trial attrition as a statistically significant difference in a measure of a PROGRESS‐Plus characteristic, between those who were and were not followed up in the intervention arm at this time point.

#### 2.5.4 Weight outcome

Weight‐related outcomes (weight change in kilograms, >5% weight loss, or change in waist circumference in centimeters) at 12‐month follow‐up were extracted. If 12‐month follow‐up data were not reported, data for the closest time point after 12–18 months of follow‐up were extracted instead. Differential weight outcome was defined as a statistically significant difference by a measure of a PROGRESS‐Plus characteristic.

#### 2.5.5 Categorization of more and less advantaged groups

We used the PROGRESS‐Plus framework, and previous inequality‐focused systematic reviews, to inform our categorization of which groups under each PROGRESS‐Plus criterion would be defined as “more advantaged” or “less advantaged.”^8^, ^28^, ^29^, ^30^ We considered “more advantaged” groups as follows: urban (place of residence, people living in urban areas often have more proximal access to healthcare and other amenities), White (race/ethnicity), employed full‐time (occupation), male (gender or sex), majority religion (religion), more education (Education), less deprived—for area based measures—or higher income level (SES), being married (social capital), and being older (PLUS). We categorized being older as “more advantaged” as evidence suggests older adults have fewer barriers to accessing primary care and are more likely to be offered weight management intervention in routine practice.^31^, ^32^, ^33^ For other measures of the plus criterion, we considered those who spoke the predominant language, were born in the country where the trial was conducted, or were free from disability to be ‘more advantaged’.

Less advantaged groups were defined as follows: rural (place of residence), ethnicity other than white (race/ethnicity), not employed full‐time (occupation), female (gender or sex), minority religion (religion), less education (education), more deprived—for area‐based measures—or lower income level (SES), not married (social capital), and being younger (PLUS). For other measures of the plus criterion, we considered those who did not speak the predominant language or were not born in the country where the trial was conducted, or who had a disability, to be “less advantaged.”

### 2.6 Quality assessment

Firstly, we extracted the categorization of quality assessment (good, fair, or poor) given to the studies included in the USPSTF report. Next, we replicated the quality assessment process from the USPSTF report for the additional studies identified as meeting eligibility criteria.^34^ Studies were graded as “good” if follow‐up was ≥80%, valid measurement instruments used, interventions clearly outlined, and confounders were appropriately controlled for in analysis. A study was rated as “fair” if some minor limitations occurred. For example, there may be minor differences in follow‐up, follow‐up <80%, or not all potential confounders accounted for. A “poor” rating was given to a study if major limitations existed, such as unreliable weight measurement methods (e.g., invalidated scales), inadequately conducted randomization, or important confounders given little or no consideration.

#### 2.6.1 Deviations from original protocol

We originally planned to use Cochrane's Risk of Bias (RoB) 2 tool to conduct risk of bias assessment.^23^ Instead, we aligned our methods for quality assessment with those used in the USPSTF report,^3^ as the USPSTF method incorporates risk of bias assessment into its quality assessment of included trials.

### 2.7 Data synthesis

Due to heterogeneity in intervention types and measures of the PROGRESS‐Plus criteria, such as country‐specific measures of SES or ethnicity, it was deemed not possible to conduct a meta‐analysis. Therefore, we conducted a narrative review with the addition of harvest plots. Harvest plots were originally proposed by Ogilvie et al., as a method of synthesizing evidence of differential effectiveness of public health interventions where a meta‐analysis may not be appropriate.^35^ In the harvest plots, bar height equates to the sample size of the study, with the smallest bars representing studies with 0–200 participants, and the tallest bars studies with 801+ participants. A study was categorized as favoring a particular group if a statistically significant difference was observed. The harvest plots were produced using Microsoft PowerPoint (version 2016, Microsoft Corporation).

## 3 RESULTS

**FIGURE 1:**
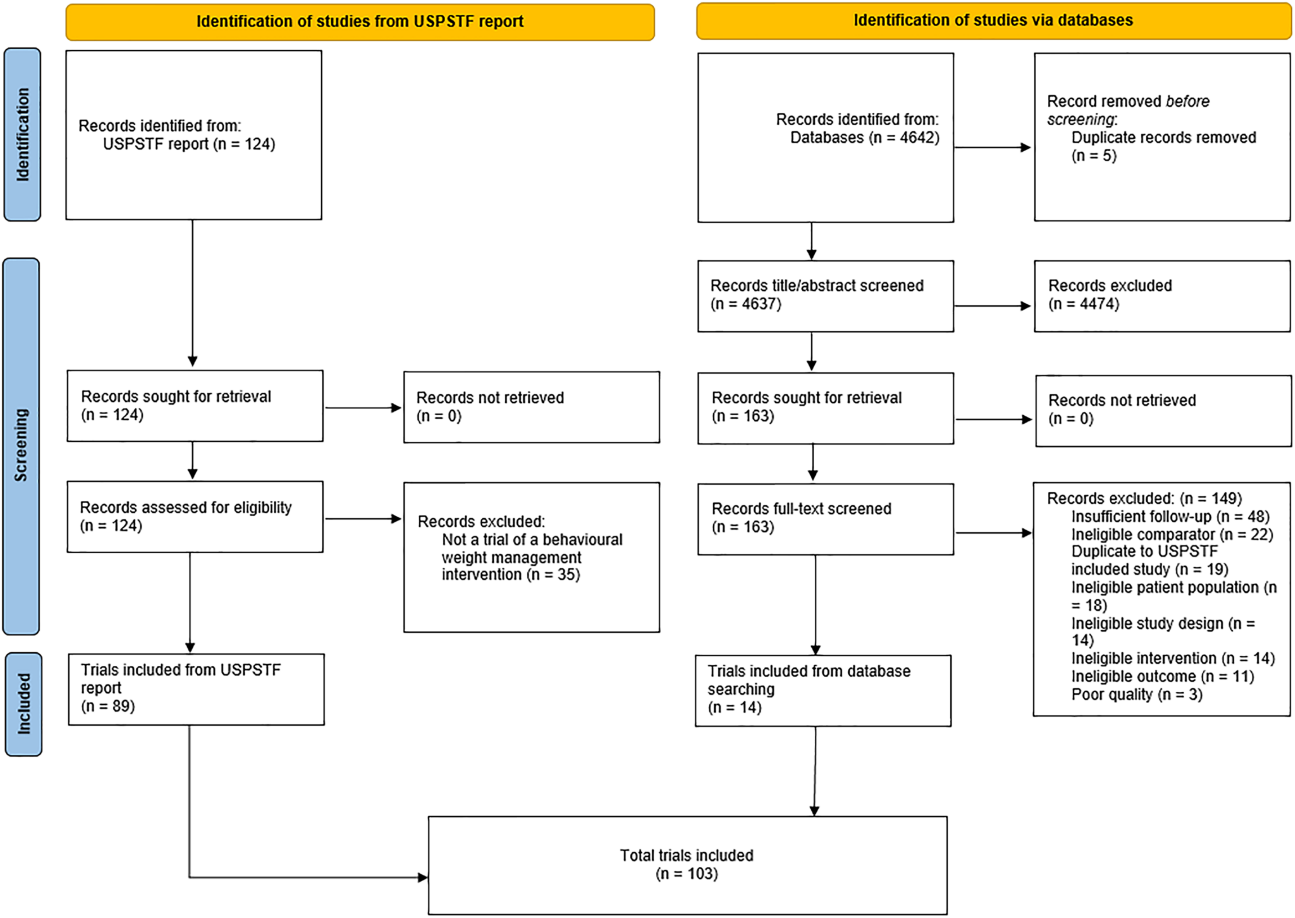
Preferred Reporting Items for Systematic Reviews and Meta‐Analysis (PRISMA) flow diagram for the inclusion of studies

The PRISMA Flow Diagram (Figure 1) shows the number of papers identified in each stage of the review.^21^ A total of 103 studies met the inclusion criteria, 89 of which were extracted from the USPSTF report and a further 14 identified through the replicated search strategy. As publication families of each included trial were identified, information was extracted from 266 publications across the 103 studies.

### 3.1 Study characteristics

Of the 103 included studies (Tables 1 and S1), 90 were trials of WLs,^36^, ^37^, ^38^, ^39^, ^40^, ^41^, ^42^, ^43^, ^44^, ^45^, ^46^, ^47^, ^48^, ^49^, ^50^, ^51^, ^52^, ^53^, ^54^, ^55^, ^56^, ^57^, ^58^, ^59^, ^60^, ^61^, ^62^, ^63^, ^64^, ^65^, ^66^, ^67^, ^68^, ^69^, ^70^, ^71^, ^72^, ^73^, ^74^, ^75^, ^76^, ^77^, ^78^, ^79^, ^80^, ^81^, ^82^, ^83^, ^84^, ^85^, ^86^, ^87^, ^88^, ^89^, ^90^, ^91^, ^92^, ^93^, ^94^, ^95^, ^96^, ^97^, ^98^, ^99^, ^100^, ^101^, ^102^, ^103^, ^104^, ^105^, ^106^, ^107^, ^108^, ^109^, ^110^, ^111^, ^112^, ^113^, ^114^, ^115^, ^116^, ^117^, ^118^, ^119^, ^120^, ^121^, ^122^, ^123^, ^124^, ^125^ and 13 were of WLMs.^126^, ^127^, ^128^, ^129^, ^130^, ^131^, ^132^, ^133^, ^134^, ^135^, ^136^, ^137^, ^138^ Across the studies, there were a total of 36,805 participants, with the sample size of each study ranging from 30^44^ to 2,161 participants.^93^ Sixty‐six percent of participants were female. Most studies were from the United States (*n* = 57).

**TABLE 1:** Characteristics of included trials

	Number of trials (%)	Citations
Intervention type		
Weight loss	90 (87.4)	^36^, ^37^, ^38^, ^39^, ^40^, ^41^, ^42^, ^43^, ^44^, ^45^, ^46^, ^47^, ^48^, ^49^, ^50^, ^51^, ^52^, ^53^, ^54^, ^55^, ^56^, ^57^, ^58^, ^59^, ^60^, ^61^, ^62^, ^63^, ^64^, ^65^, ^66^, ^67^, ^68^, ^69^, ^70^, ^71^, ^72^, ^73^, ^74^, ^75^, ^76^, ^77^, ^78^, ^79^, ^80^, ^81^, ^82^, ^83^, ^84^, ^85^, ^86^, ^87^, ^88^, ^89^, ^90^, ^91^, ^92^, ^93^, ^94^, ^95^, ^96^, ^97^, ^98^, ^99^, ^100^, ^101^, ^102^, ^103^, ^104^, ^105^, ^106^, ^107^, ^108^, ^109^, ^110^, ^111^, ^112^, ^113^, ^114^, ^115^, ^116^, ^117^, ^118^, ^119^, ^120^, ^121^, ^122^, ^123^, ^124^, ^125^
Weight loss maintenance	13 (12.6)	^126^, ^127^, ^128^, ^129^, ^130^, ^131^, ^132^, ^133^, ^134^, ^135^, ^136^, ^137^, ^138^
Database searching vs. identification from USPSTF report		
Studies identified from database searching	14 (13.6)	^84^, ^114^, ^115^, ^116^, ^117^, ^118^, ^119^, ^120^, ^121^, ^122^, ^135^, ^136^, ^137^, ^138^
Studies identified from USPSTF report	89 (86.4)	^36^, ^37^, ^38^, ^39^, ^40^, ^41^, ^42^, ^43^, ^44^, ^45^, ^46^, ^47^, ^48^, ^49^, ^50^, ^51^, ^52^, ^53^, ^54^, ^55^, ^56^, ^57^, ^58^, ^59^, ^60^, ^61^, ^62^, ^63^, ^64^, ^65^, ^66^, ^67^, ^68^, ^69^, ^70^, ^71^, ^72^, ^73^, ^74^, ^75^, ^76^, ^77^, ^78^, ^79^, ^80^, ^81^, ^82^, ^83^, ^85^, ^86^, ^87^, ^88^, ^89^, ^90^, ^91^, ^92^, ^93^, ^94^, ^95^, ^96^, ^97^, ^98^, ^99^, ^100^, ^101^, ^102^, ^103^, ^104^, ^105^, ^106^, ^107^, ^108^, ^109^, ^110^, ^111^, ^112^, ^113^, ^123^, ^124^, ^125^, ^126^, ^127^, ^128^, ^129^, ^130^, ^131^, ^132^, ^133^, ^134^
Trial location		
United States	57 (55.3)	^36^, ^37^, ^42^, ^43^, ^44^, ^45^, ^46^, ^47^, ^52^, ^55^, ^56^, ^57^, ^63^, ^64^, ^65^, ^66^, ^68^, ^69^, ^71^, ^73^, ^74^, ^75^, ^76^, ^77^, ^78^, ^79^, ^80^, ^81^, ^83^, ^84^, ^85^, ^87^, ^90^, ^92^, ^93^, ^94^, ^96^, ^98^, ^101^, ^102^, ^104^, ^105^, ^107^, ^110^, ^111^, ^112^, ^113^, ^115^, ^117^, ^119^, ^123^, ^127^, ^129^, ^130^, ^131^, ^133^, ^134^
United Kingdom	16 (15.5)	^38^, ^39^, ^40^, ^41^, ^48^, ^49^, ^53^, ^61^, ^67^, ^95^, ^100^, ^114^, ^124^, ^132^, ^135^, ^138^
Australia	5 (4.9)	^59^, ^116^, ^118^, ^125^, ^128^
Finland	5 (4.9)	^86^, ^103^, ^108^, ^122^, ^126^
Japan	4 (3.9)	^60^, ^91^, ^99^, ^137^
Germany	3 (2.9)	^54^, ^97^, ^136^
The Netherlands	3 (2.9)	^58^, ^82^, ^109^
Spain	3 (2.9)	^106^, ^120^, ^121^
Canada	2 (1.9)	^70^, ^89^
Sweden	2 (1.9)	^50^, ^88^
Norway	1 (1.0)	^62^
Portugal	1 (1.0)	^72^
Multiple countries	1 (1.0)	^51^
Sample size at baseline		
0–200	41 (39.8)	^36^, ^44^, ^45^, ^46^, ^48^, ^50^, ^54^, ^57^, ^58^, ^59^, ^64^, ^65^, ^67^, ^71^, ^76^, ^80^, ^81^, ^86^, ^88^, ^90^, ^91^, ^97^, ^99^, ^101^, ^102^, ^103^, ^104^, ^107^, ^110^, ^112^, ^113^, ^119^, ^120^, ^121^, ^124^, ^128^, ^129^, ^130^, ^132^, ^136^, ^137^
201–400	32 (31.1)	^39^, ^42^, ^43^, ^47^, ^52^, ^55^, ^56^, ^60^, ^61^, ^62^, ^63^, ^68^, ^69^, ^72^, ^75^, ^77^, ^83^, ^84^, ^87^, ^92^, ^98^, ^111^, ^114^, ^115^, ^116^, ^117^, ^118^, ^125^, ^126^, ^133^, ^134^, ^138^
401–600	15 (14.6)	^37^, ^41^, ^66^, ^70^, ^73^, ^78^, ^79^, ^82^, ^85^, ^94^, ^108^, ^122^, ^123^, ^131^, ^135^
601–800	5 (4.9)	^49^, ^51^, ^53^, ^96^, ^105^
>800	10 (9.7)	^38^, ^40^, ^74^, ^89^, ^93^, ^95^, ^100^, ^106^, ^109^, ^127^
Proportion female at baseline		
0% (all male)	6 (5.8)	^49^, ^59^, ^66^, ^103^, ^109^, ^128^
1–49%	11 (10.7)	^39^, ^48^, ^54^, ^56^, ^58^, ^73^, ^74^, ^91^, ^97^, ^119^, ^133^
50–99%	70 (68.0)	^36^, ^37^, ^38^, ^40^, ^41^, ^42^, ^43^, ^44^, ^46^, ^51^, ^52^, ^53^, ^55^, ^60^, ^61^, ^62^, ^63^, ^64^, ^65^, ^67^, ^69^, ^70^, ^71^, ^75^, ^76^, ^77^, ^78^, ^79^, ^81^, ^83^, ^84^, ^85^, ^86^, ^87^, ^88^, ^89^, ^90^, ^92^, ^93^, ^95^, ^96^, ^98^, ^99^, ^100^, ^106^, ^107^, ^108^, ^111^, ^112^, ^113^, ^114^, ^115^, ^116^, ^118^, ^120^, ^121^, ^122^, ^123^, ^124^, ^125^, ^126^, ^127^, ^130^, ^131^, ^132^, ^134^, ^135^, ^136^, ^137^, ^138^
100% (all female)	16 (15.5)	^45^, ^47^, ^50^, ^57^, ^68^, ^72^, ^80^, ^82^, ^94^, ^101^, ^102^, ^104^, ^105^, ^110^, ^117^, ^129^
Mean age (years) at baseline^a^		
18–40	11 (10.7)	^50^, ^52^, ^59^, ^68^, ^72^, ^75^, ^76^, ^85^, ^86^, ^101^, ^117^
41–54	63 (61.2)	^37^, ^38^, ^42^, ^43^, ^45^, ^46^, ^47^, ^49^, ^51^, ^53^, ^55^, ^56^, ^57^, ^60^, ^61^, ^62^, ^63^, ^64^, ^65^, ^66^, ^69^, ^70^, ^71^, ^73^, ^74^, ^79^, ^81^, ^83^, ^84^, ^87^, ^88^, ^89^, ^93^, ^95^, ^96^, ^97^, ^98^, ^100^, ^102^, ^103^, ^104^, ^107^, ^109^, ^111^, ^112^, ^114^, ^115^, ^116^, ^118^, ^119^, ^121^, ^122^, ^123^, ^124^, ^126^, ^128^, ^129^, ^130^, ^131^, ^132^, ^134^, ^135^, ^136^, ^138^
≥55	28 (27.2)	^36^, ^39^, ^40^, ^41^, ^44^, ^48^, ^54^, ^58^, ^67^, ^77^, ^78^, ^80^, ^82^, ^90^, ^91^, ^92^, ^94^, ^99^, ^105^, ^106^, ^108^, ^110^, ^113^, ^120^, ^125^, ^127^, ^133^, ^137^
Number of PROGRESS‐Plus measures reported at baseline		
0	0 (0.0)	
1	0 (0.0)	
2	20	^44^, ^46^, ^58^, ^60^, ^70^, ^88^, ^97^, ^99^, ^103^, ^106^, ^108^, ^112^, ^121^, ^129^, ^130^, ^131^, ^134^, ^136^, ^137^
3	16	^36^, ^43^, ^51^, ^54^, ^85^, ^87^, ^90^, ^91^, ^95^, ^96^, ^100^, ^105^, ^107^, ^113^, ^122^, ^132^
4+	67	^37^, ^38^, ^39^, ^40^, ^41^, ^42^, ^45^, ^47^, ^48^, ^49^, ^50^, ^52^, ^53^, ^55^, ^56^, ^57^, ^59^, ^61^, ^62^, ^63^, ^64^, ^65^, ^66^, ^67^, ^68^, ^69^, ^71^, ^72^, ^73^, ^74^, ^75^, ^76^, ^77^, ^78^, ^79^, ^80^, ^81^, ^82^, ^83^, ^84^, ^86^, ^89^, ^92^, ^93^, ^94^, ^98^, ^101^, ^102^, ^104^, ^109^, ^110^, ^111^, ^114^, ^115^, ^116^, ^117^, ^118^, ^119^, ^120^, ^123^, ^124^, ^126^, ^127^, ^128^, ^133^, ^135^, ^138^
Number of PROGRESS‐Plus measures differential trial uptake considered by		
0	89 (86.4)	^36^, ^37^, ^39^, ^41^, ^43^, ^44^, ^46^, ^47^, ^49^, ^50^, ^51^, ^52^, ^53^, ^54^, ^55^, ^56^, ^57^, ^59^, ^60^, ^61^, ^62^, ^63^, ^64^, ^65^, ^67^, ^69^, ^72^, ^73^, ^75^, ^76^, ^77^, ^78^, ^80^, ^81^, ^82^, ^83^, ^84^, ^85^, ^86^, ^87^, ^88^, ^89^, ^90^, ^91^, ^92^, ^93^, ^94^, ^95^, ^96^, ^97^, ^98^, ^99^, ^100^, ^101^, ^102^, ^103^, ^104^, ^105^, ^106^, ^107^, ^108^, ^109^, ^110^, ^111^, ^112^, ^113^, ^114^, ^115^, ^116^, ^117^, ^118^, ^119^, ^120^, ^121^, ^122^, ^123^, ^124^, ^125^, ^126^, ^129^, ^130^, ^131^, ^132^, ^135^, ^136^, ^137^, ^138^
1	6 (5.8)	^48^, ^58^, ^68^, ^70^, ^71^, ^119^
2	4 (3.9)	^42^, ^45^, ^66^, ^114^
3	3 (2.9)	^40^, ^74^, ^143^
4+	1 (1.0)	^79^
Number of PROGRESS‐Plus measures differential intervention uptake considered by		
0	98 (95.1)	^36^, ^38^, ^39^, ^41^, ^42^, ^43^, ^44^, ^45^, ^46^, ^47^, ^48^, ^49^, ^50^, ^51^, ^53^, ^54^, ^55^, ^56^, ^57^, ^58^, ^59^, ^60^, ^61^, ^62^, ^63^, ^64^, ^65^, ^66^, ^67^, ^69^, ^70^, ^71^, ^72^, ^73^, ^74^, ^75^, ^76^, ^77^, ^78^, ^79^, ^80^, ^81^, ^82^, ^83^, ^84^, ^85^, ^86^, ^87^, ^88^, ^89^, ^90^, ^91^, ^92^, ^93^, ^94^, ^95^, ^96^, ^97^, ^98^, ^99^, ^100^, ^101^, ^102^, ^103^, ^104^, ^105^, ^106^, ^107^, ^108^, ^109^, ^110^, ^111^, ^112^, ^113^, ^114^, ^115^, ^116^, ^117^, ^118^, ^119^, ^120^, ^121^, ^122^, ^123^, ^124^, ^125^, ^126^, ^127^, ^128^, ^129^, ^130^, ^131^, ^132^, ^133^, ^134^, ^135^, ^136^, ^137^, ^138^
1	3 (2.9)	^40^, ^52^, ^68^
2	0 (0.0)	
3	0 (0.0)	
4+	1 (1.0)	^37^
Number of PROGRESS‐Plus measures differential adherence considered by		
0	88 (85.4)	^36^, ^39^, ^41^, ^43^, ^44^, ^45^, ^46^, ^47^, ^48^, ^49^, ^50^, ^51^, ^53^, ^54^, ^56^, ^57^, ^58^, ^60^, ^61^, ^62^, ^63^, ^64^, ^65^, ^66^, ^67^, ^69^, ^72^, ^74^, ^75^, ^77^, ^78^, ^79^, ^80^, ^81^, ^82^, ^83^, ^85^, ^86^, ^87^, ^88^, ^89^, ^90^, ^91^, ^92^, ^93^, ^94^, ^95^, ^96^, ^97^, ^98^, ^99^, ^100^, ^101^, ^102^, ^103^, ^104^, ^105^, ^106^, ^107^, ^108^, ^109^, ^110^, ^111^, ^112^, ^113^, ^115^, ^116^, ^117^, ^118^, ^120^, ^121^, ^122^, ^123^, ^124^, ^125^, ^126^, ^128^, ^129^, ^130^, ^131^, ^132^, ^133^, ^134^, ^135^, ^136^, ^137^, ^138^
1	7 (6.8)	^40^, ^52^, ^55^, ^68^, ^70^, ^73^, ^76^
2	1 (1.0)	^59^
3	2 (1.9)	^71^, ^114^
4+	5 (4.9)	^37^, ^38^, ^42^, ^119^, ^127^
Number of PROGRESS‐Plus measures differential trial attrition considered by		
0	71 (68.9)	^37^, ^39^, ^40^, ^43^, ^46^, ^48^, ^49^, ^55^, ^56^, ^58^, ^60^, ^64^, ^73^, ^74^, ^75^, ^76^, ^78^, ^80^, ^81^, ^82^, ^83^, ^85^, ^86^, ^87^, ^88^, ^89^, ^90^, ^91^, ^92^, ^93^, ^94^, ^95^, ^96^, ^97^, ^98^, ^99^, ^100^, ^101^, ^102^, ^103^, ^104^, ^105^, ^106^, ^107^, ^108^, ^109^, ^110^, ^111^, ^112^, ^113^, ^123^, ^124^, ^125^, ^126^, ^129^, ^130^, ^131^, ^132^, ^133^, ^134^
1	6 (5.8)	^51^, ^52^, ^59^, ^63^, ^67^, ^69^
2	7 (6.8)	^44^, ^50^, ^62^, ^65^, ^66^, ^70^, ^117^
3	7 (6.8)	^36^, ^41^, ^54^, ^57^, ^71^, ^72^, ^114^
4+	12 (11.7)	^38^, ^42^, ^45^, ^47^, ^53^, ^61^, ^68^, ^77^, ^79^, ^116^, ^127^, ^135^
Number of PROGRESS‐Plus measures differential weight outcome considered by		
0	72 (69.9)	^36^, ^37^, ^41^, ^45^, ^47^, ^48^, ^50^, ^51^, ^54^, ^55^, ^57^, ^58^, ^60^, ^62^, ^63^, ^64^, ^65^, ^66^, ^67^, ^70^, ^71^, ^72^, ^76^, ^77^, ^80^, ^81^, ^82^, ^83^, ^85^, ^86^, ^87^, ^88^, ^89^, ^90^, ^91^, ^92^, ^94^, ^95^, ^96^, ^97^, ^98^, ^99^, ^100^, ^101^, ^102^, ^103^, ^104^, ^105^, ^106^, ^107^, ^108^, ^109^, ^110^, ^111^, ^112^, ^113^, ^119^, ^120^, ^121^, ^122^, ^123^, ^124^, ^125^, ^129^, ^130^, ^131^, ^132^, ^133^, ^134^, ^136^, ^137^, ^138^
1	10 (9.7)	^40^, ^46^, ^52^, ^53^, ^56^, ^59^, ^61^, ^69^, ^116^, ^118^
2	8 (7.8)	^44^, ^73^, ^78^, ^79^, ^93^, ^115^, ^117^, ^135^
3	7 (6.8)	^38^, ^39^, ^43^, ^74^, ^114^, ^126^, ^128^, ^143^
4+	6 (5.8)	^42^, ^49^, ^68^, ^75^, ^84^, ^127^

Abbreviations: PROGRESS‐Plus, place of residence, race/ethnicity, occupation, gender, religion, education, socioeconomic status, social capital, plus other discriminating factors; USPSTF, United States Preventive Services Taskforce.

^a^ Mean age not available for Perri et al.^130^

Thirty‐six of the included studies explicitly stated that the intervention targeted a specific group that covered at least one of the PROGRESS‐Plus criteria.^37^, ^39^, ^42^, ^43^, ^45^, ^47^, ^49^, ^50^, ^57^, ^59^, ^62^, ^63^, ^65^, ^66^, ^68^, ^69^, ^72^, ^75^, ^76^, ^78^, ^80^, ^81^, ^82^, ^85^, ^94^, ^102^, ^103^, ^104^, ^105^, ^109^, ^110^, ^113^, ^117^, ^119^, ^124^, ^129^ Specifically, 10 studies targeted a specific race or ethnic group (such as Black African‐American or Hispanic),^43^, ^47^, ^57^, ^63^, ^65^, ^69^, ^76^, ^102^, ^113^, ^124^ three targeted specific occupations,^103^, ^109^, ^119^ 22 studies targeted based on gender or sex (six interventions were targeted at men^49^, ^59^, ^66^, ^103^, ^109^, ^128^ and 16 at women^45^, ^47^, ^50^, ^57^, ^68^, ^72^, ^80^, ^82^, ^94^, ^101^, ^102^, ^104^, ^105^, ^110^, ^117^, ^129^), eight targeted low‐income groups,^37^, ^42^, ^57^, ^65^, ^68^, ^69^, ^81^, ^102^ and five targeted particular age groups.^39^, ^75^, ^78^, ^85^, ^129^ Participants belonging to certain health groups were also targeted in eight of the studies identified,^39^, ^45^, ^50^, ^62^, ^68^, ^80^, ^105^, ^110^ such as postpartum women or those at elevated risk of breast cancer.

### 3.2 Quality assessment

As shown in Figure 1, we scored three studies as “poor” quality; these were excluded from our synthesis.^139^, ^140^, ^141^ Of the 14 studies identified in our updated search, 11 were scored “fair” quality, and three were scored “good” quality. In total, 74 studies were of “fair” quality, and 29 of “good” quality (Table S1).

### 3.3 Findings

**FIGURE 2:**
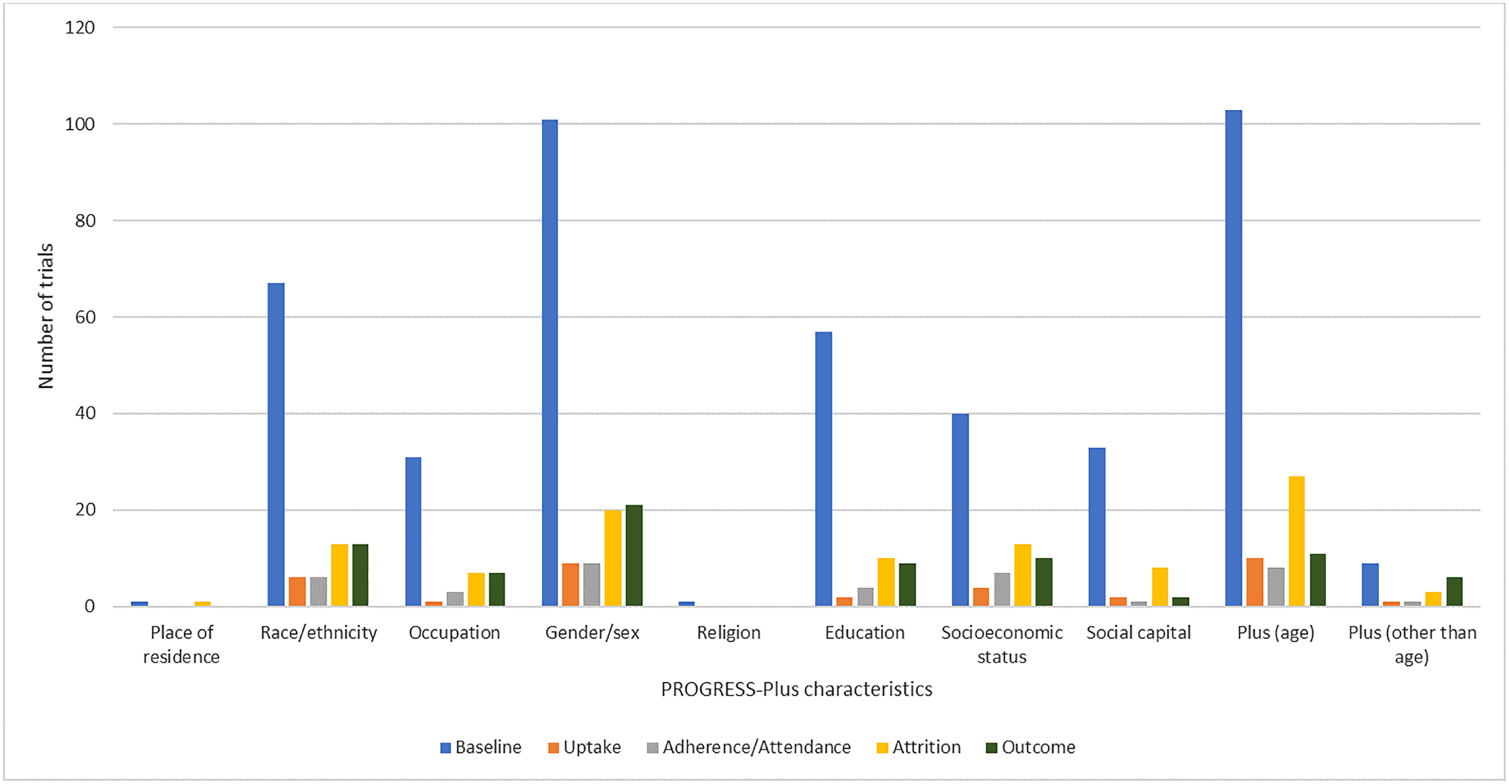
Number of trials reporting PROGRESS‐Plus (place of residence, race/ethnicity, occupation, gender, religion, education, socioeconomic status, social capital, plus other discriminating factors) criteria at each trial stage

At baseline, all 103 trials reported age and almost all (*n* = 101) reported participant gender or sex (Figure 2). The next most commonly reported baseline measures were race/ethnicity (*n* = 67), education (*n* = 57), SES (*n* = 40), social capital (predominantly marital status, *n* = 33), and occupation (*n* = 31). Nine trials reported measures (other than age) that meet the definition of “plus” according to the PROGRESS‐Plus characteristics—the most common measures meeting this criterion was health literacy and language at home. The least commonly reported measures at baseline were place of residence (*n* = 1) and religion (*n* = 1). Sexual orientation was not reported in any of the included studies. Fifty‐six of the 103 trials considered inequalities in intervention or trial uptake (*n* = 15), intervention adherence (*n* = 15), trial attrition (*n* = 32), or weight outcome (*n* = 34) in relation to at least one PROGRESS‐Plus criteria.

#### 3.3.1 Inequalities and uptake

##### Trial uptake

**FIGURE 3:**
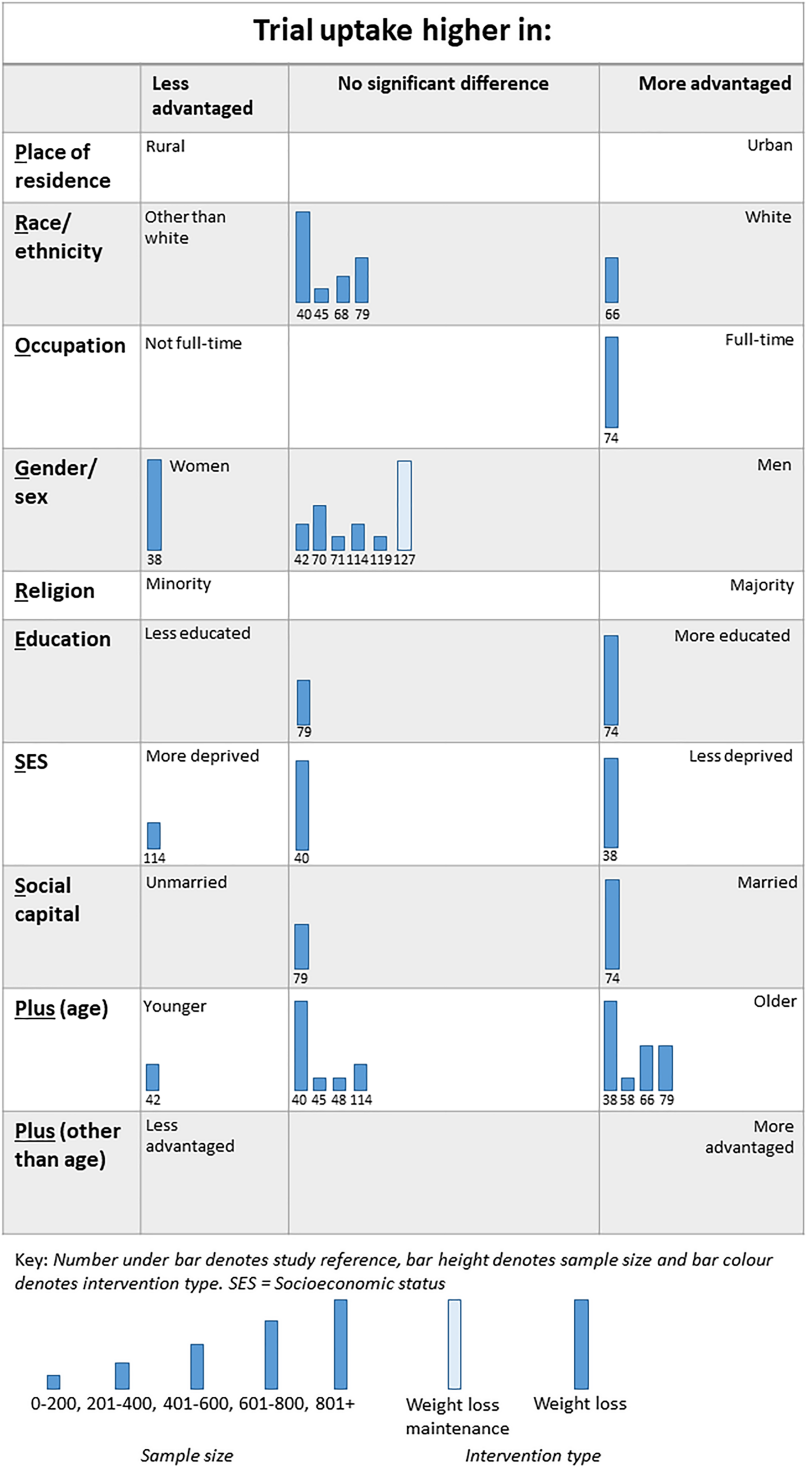
Harvest plot of inequalities in trial uptake

Twenty‐nine analyses (WLs = 28, WLMs = 1) across 15 trials (Figure 3) examined inequalities in trial uptake.^38^, ^40^, ^42^, ^45^, ^48^, ^58^, ^66^, ^68^, ^70^, ^71^, ^74^, ^79^, ^114^, ^119^, ^127^

In the 28 analyses across 14 trials of WLs, 16 analyses found no evidence that trial uptake favored more or less advantaged. Three analyses found that trial uptake was highest in “less advantaged” groups and nine analyses found trial uptake was highest in “more advantaged” groups.

One study (one analysis) considered if differential trial uptake occurred in WLMs trials. This analysis found no evidence that trial uptake favored more or less advantaged.

##### Intervention uptake

**FIGURE 4:**
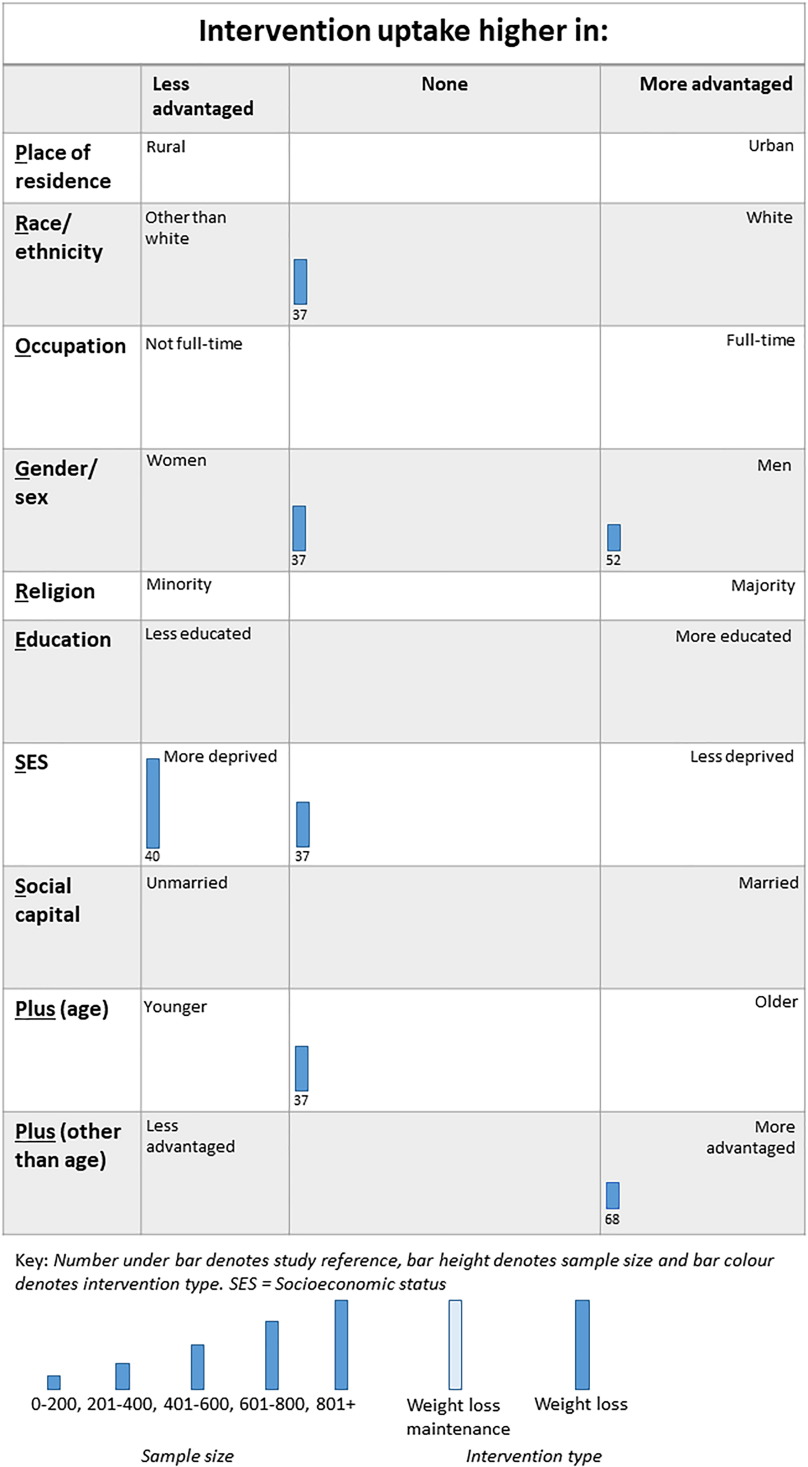
Harvest plot of inequalities in intervention uptake

Seven analyses across four trials (all WLs) considered whether there were inequalities in intervention uptake (Figure 4).^37^, ^40^, ^66^, ^73^ One study considered inequalities by race or ethnicity, two studies considered inequalities by gender, one by SES, one by social capital (marital status), one by age, and one by protocol language (English versus Spanish). One analysis found that intervention uptake favored “less advantaged,”^40^ three analyses found no gradient,^37^ two found intervention uptake favored “more advantaged,”^66^ while one analysis was unclear in whether it favored a particular group or not.^73^

#### 3.3.2 Inequalities and intervention adherence

**FIGURE 5:**
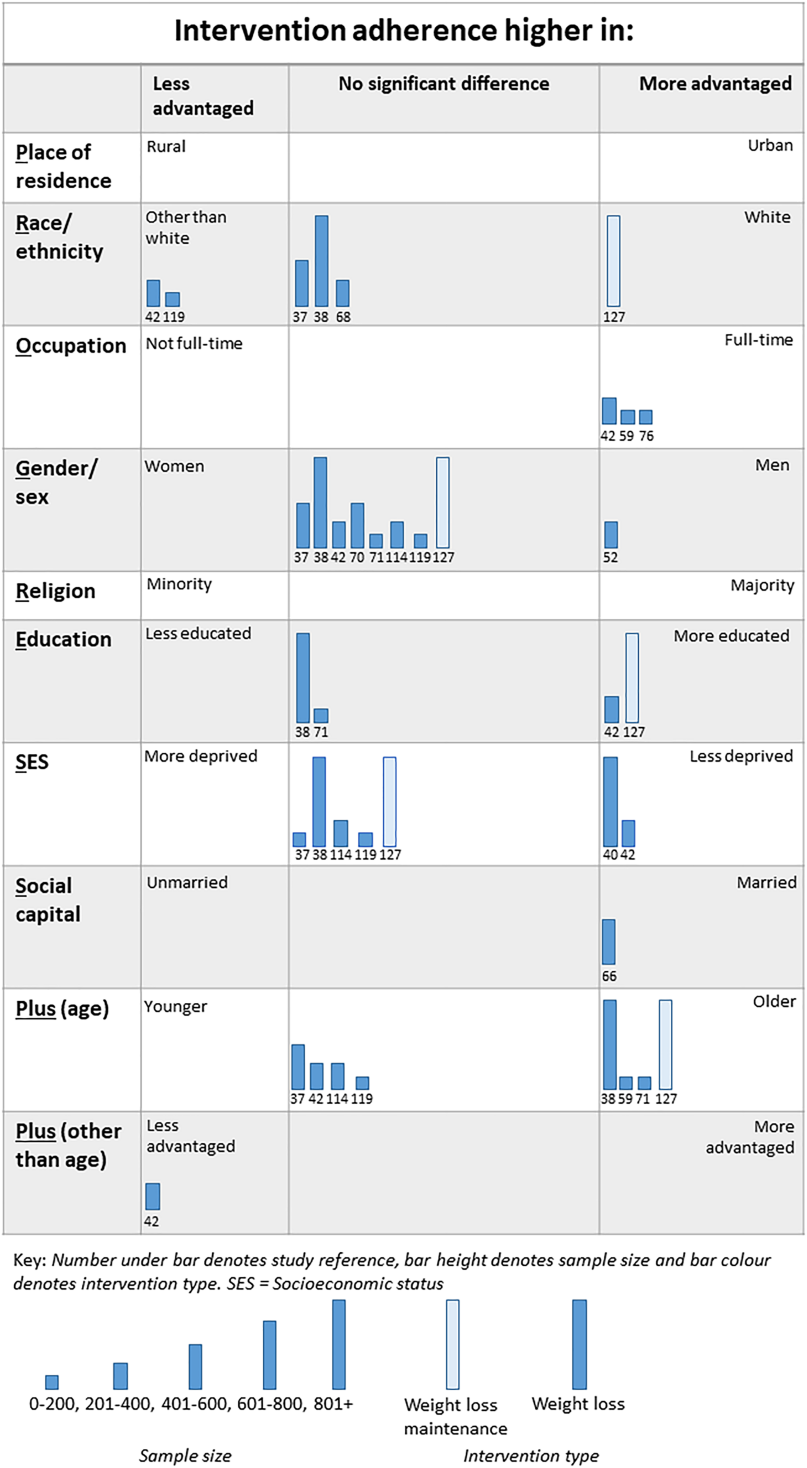
Harvest plot of inequalities in adherence to behavioral weight management interventions

Thirty‐nine analyses (WLs = 34, WLMs = 5) from 15 trials (Figure 5) examined inequalities in intervention adherence.

We found 34 analyses across 14 trials of WLs that examined inequalities in intervention adherence.^37^, ^38^, ^40^, ^42^, ^52^, ^55^, ^59^, ^68^, ^70^, ^71^, ^73^, ^76^, ^114^, ^119^ Twenty of the 34 analyses found no gradient. Eleven analyses found that intervention adherence favored more advantaged groups (i.e., that intervention adherence was highest in these groups) and three found that intervention adherence was highest in the less advantaged groups. Intervention adherence was higher in those who had a full‐time occupation versus not full‐time (3/3 analyses) and also appeared to be higher in older participants (3/7 analyses).

Five analyses, from one trial, explored inequalities in adherence to WLMs.^127^ Three out of the five analyses favored the more advantaged groups (1/1 analysis of ethnicity, 1/1 analysis of education, and 1/1 analysis of age). The remaining two analyses found that intervention adherence did not favor either less or more advantaged groups.

#### 3.3.3 Inequalities and trial attrition

**FIGURE 6:**
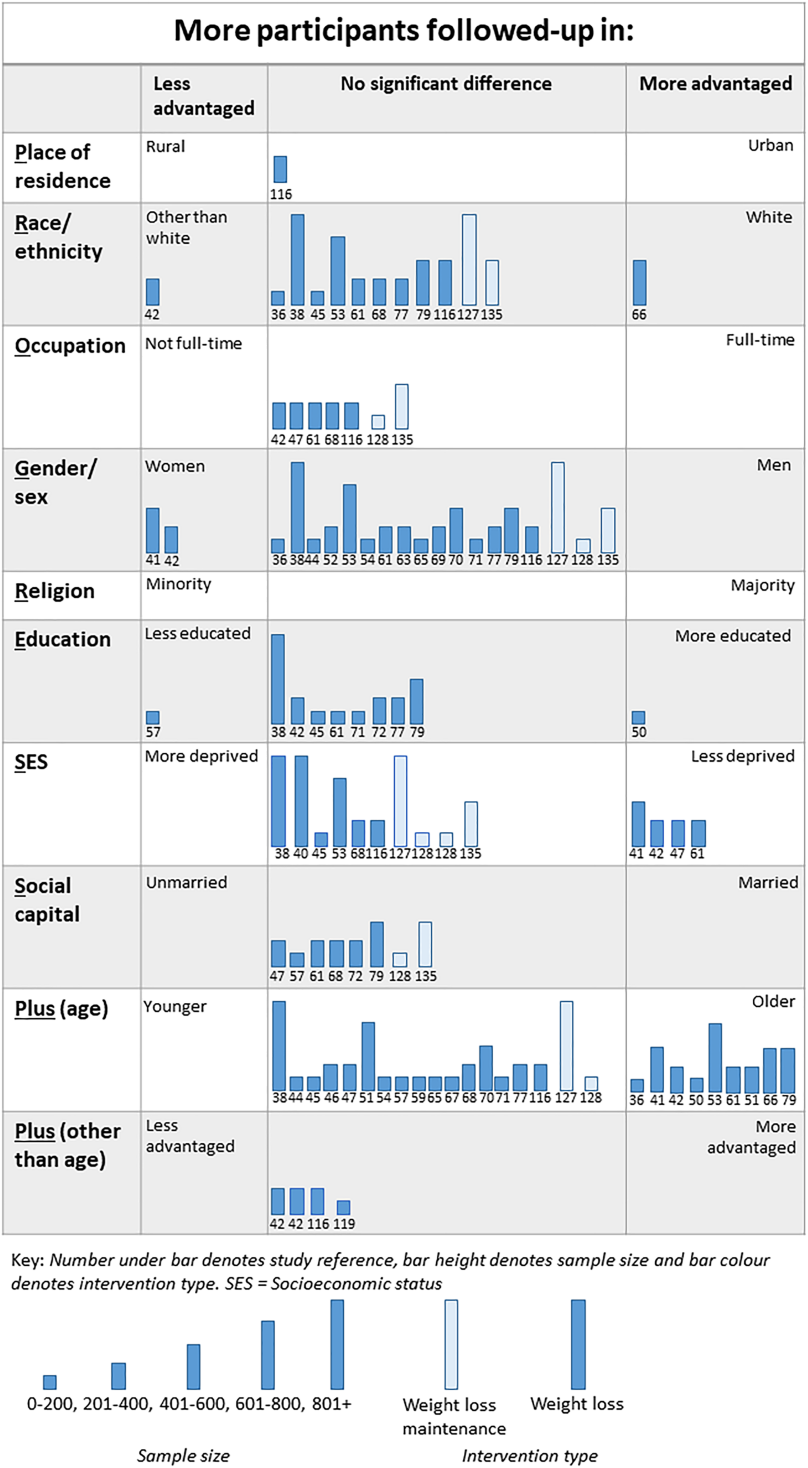
Harvest plot of inequalities in trial attrition

In total, 93 (WLs = 78, WLMs = 15) analyses across 32 trials considered inequalities in trial attrition (Figure 6).

Seventy‐eight analyses from 29 trials assessed inequalities in trial attrition in WLs.^36^, ^38^, ^41^, ^42^, ^44^, ^45^, ^47^, ^50^, ^51^, ^52^, ^53^, ^54^, ^57^, ^59^, ^61^, ^62^, ^63^, ^65^, ^66^, ^67^, ^68^, ^69^, ^70^, ^71^, ^72^, ^77^, ^79^, ^114^, ^116^ The majority of these analyses (*n* = 59) found that trial attrition favored no particular group; four analyses found that trial attrition was lower in less advantaged groups, and in 15 analyses, trial attrition was lower in more advantaged groups. Most of the analyses favoring “more advantaged” were of age, followed by SES (i.e., those who were older or of a less deprived SES were less likely to be lost to follow‐up). There was little evidence to suggest inequalities in trial attrition by other PROGRESS‐Plus criteria in trial attrition.

All analyses (15 across three trials) considering if there were inequalities in trial attrition in WLMs found that trial attrition did not favor any particular group.^127^, ^128^, ^135^

#### 3.3.4 Inequalities and weight outcome

**FIGURE 7:**
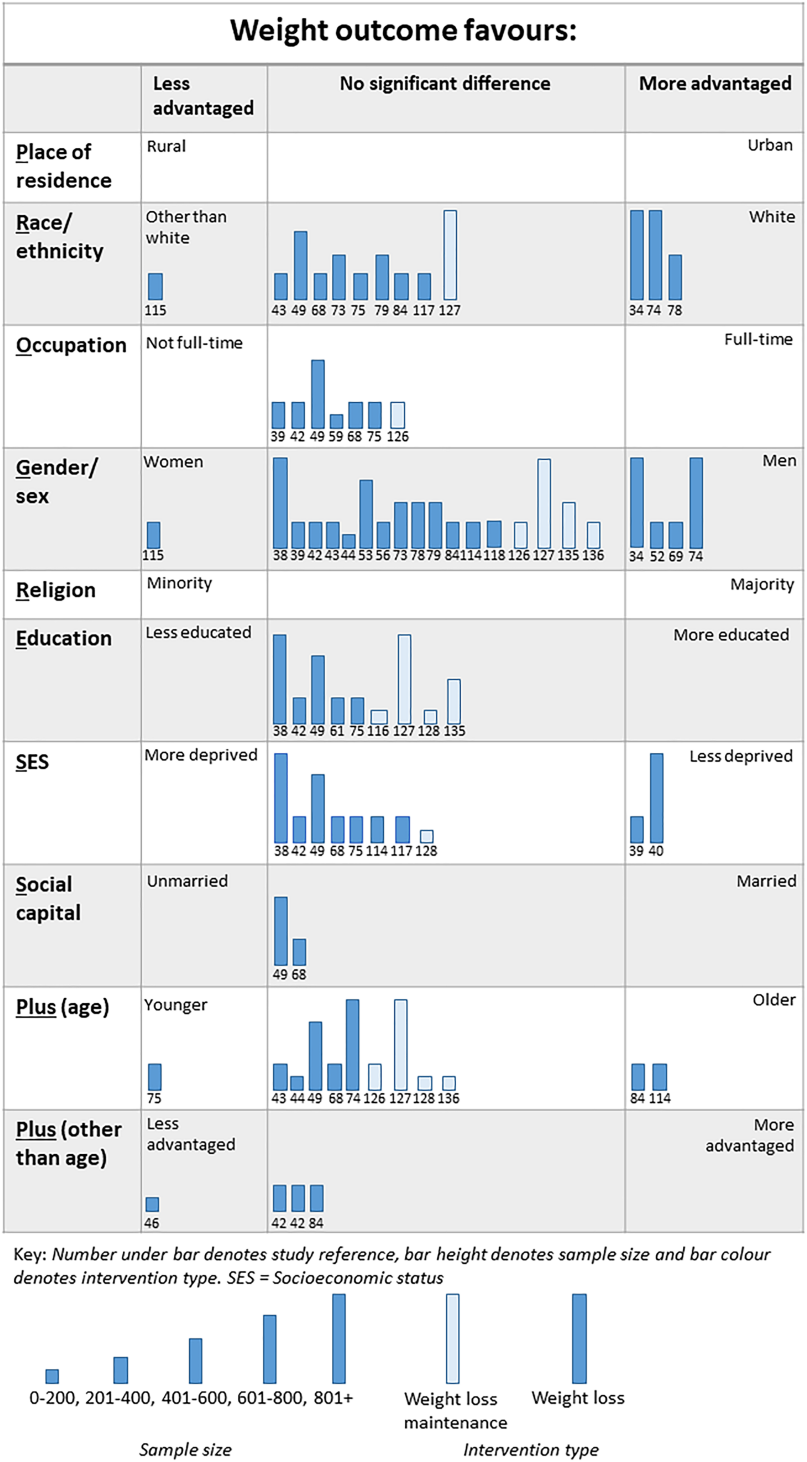
Harvest plot of inequalities in weight outcome

We identified 79 analyses (WLs = 64, WLMs = 15) across 34 trials that considered inequalities in weight outcome (Figure 7). The results of four of these analyses (three for gender or sex^60^, ^64^, ^70^ and one for occupation^126^) were unclear and are consequently omitted from the Harvest plot.

Sixty‐five analyses in 30 trials of WLs considered inequalities in weight loss.^36^, ^37^, ^38^, ^39^, ^40^, ^42^, ^43^, ^44^, ^46^, ^49^, ^52^, ^53^, ^56^, ^59^, ^60^, ^64^, ^68^, ^69^, ^70^, ^73^, ^74^, ^75^, ^78^, ^79^, ^84^, ^93^, ^114^, ^115^, ^117^, ^118^ Four analyses found that less advantaged groups lost more weight, 11 that more advantaged groups lost more weight, and the majority (*n* = 50) found that weight loss favored neither less nor more advantaged groups. For gender or sex, men lost more weight than women in three out of 17 analyses conducted, whereas women lost more weight in one of the 17 analyses. For SES, two of nine analyses favored those in less advantaged groups.

We identified 15 analyses of inequalities in weight loss maintenance across five WLMs.^126^, ^127^, ^128^, ^135^, ^136^ None of the analyses found the inequalities in the weight outcome of WLMs (i.e., there was no significant difference observed in weight loss maintenance by any measure of inequality).

## 4 DISCUSSION

This comprehensive systematic review found that most trials of behavioral weight management interventions do not examine whether differential trial/intervention uptake, intervention adherence, trial attrition, or outcome occurs in different social groups. In those that did examine differences, most found no gradient (e.g., intervention uptake or trial attrition was not higher in either more or less advantaged groups). Where a gradient was observed, it mostly favored those who were “more advantaged.” This was not the case for weight outcomes, for which a similar number of trials favored “less advantaged” groups as those favoring “more advantaged.” Our findings suggest that inequalities may occur in intervention/trial uptake, intervention adherence, and trial attrition, although behavioral weight management interventions may be equitable for those who reach the 12‐month follow‐up.

In this review, we examined two types of behavioral weight management interventions: interventions targeting weight loss (WLs) and interventions targeting weight loss maintenance (WLMs). There were differences in inequalities observed between the two intervention types; evidence of inequalities in WLMs was only present in intervention adherence and not in trial or intervention uptake, whereas there was some evidence of inequalities at all stages in WLs. There may be several underpinning reasons for this. First, we identified fewer trials of WLMs than of WLs (13 vs. 90), meaning that there were fewer data available for WLMs. Second, it is possible that inequalities in behavioral weight management interventions are being generated in the interventions targeting weight loss, and those who are less successful in a weight loss intervention may be less likely to be invited to take part in a further weight loss maintenance trial.

We found some evidence to suggest, when taking into account the age of people invited to take part in a weight management trial, a significantly higher proportion of older people took up the offer in four of the 10 studies we identified that examined this. This is supported by survey data showing that older people report better access to primary care^142^ and by evidence from a UK‐based population‐based cohort study which observed that weight management interventions were more often accessed by older participants.^33^ The study authors also noted that weight management interventions were more often accessed by women and those in deprivation.^33^ We found that, overall, two thirds of total participants across the trials included in our review were female. This is similar to the findings of previous systematic review which focused on the issue of male inclusion in RCTs in WLs, which found that only 27% of participants were male.^16^ When accounting for the total number of men and women invited to take part in the trial, we did not find evidence to suggest there were inequalities in those who were likely to accept invitation to the trial or to the intervention arm. It has previously been observed that the proportion of male participants in studies of commercial weight management programs is higher when all eligible in the population are invited than when patients are invited opportunistically, suggesting the inequality in male participation can be reduced by inviting more men to take part.^143^

When compared with the wider RCT literature, trials of behavioral weight management interventions are atypical in that recruitment favors women and older participants. Outside of trials of behavioral weight management interventions, for example, in RCTs used for cardiovascular guidelines or drug and vaccine research, recruitment tends to favor men and younger participants.^144^, ^145^, ^146^ Therefore, this may suggest that a nuanced perspective of inequalities should be taken when addressing behavioral weight management interventions, as some groups that are typically underresearched—women and older people—are the most researched. Hence, there is less research in groups that would typically be considered as advantaged in other health and wider societal domains.

Although there is some evidence in our systematic review to suggest that trial uptake is higher in less socioeconomically advantaged groups, we found that intervention adherence, trial attrition, and weight outcome favored those who were less deprived. This supports findings from trials of other behavioral interventions (such as those targeting smoking cessation) where attrition is higher or intervention/adherence is lower in those who are more deprived.^147^, ^148^, ^149^, ^150^ Future studies should consider how participants from more deprived backgrounds can remain engaged in both the intervention and the trial itself, to ensure that the benefit of the intervention can be received more equally across different socioeconomic groups. For example, incentives may have an important role in improving participation in trials of health interventions, particularly in groups that are typically underrepresented. However, due to a lack of relevant data reported in the included studies, we have been unable to examine this.

Our findings of inequalities in behavioral weight management interventions by race or ethnicity are broadly similar to previously conducted systematic reviews we identified. We found that few studies reported if there was differential adherence or outcome by race or ethnicity, supporting the findings of Tussing‐Humphreys et al. and Haughton et al.^13^, ^15^ We did not have sufficient evidence to support Tussing‐Humphreys et al. and Kong et al. findings that WL and WLM led to less weight loss and more weight regain in African American women though.^10^, ^15^ This may have been due to the different inclusion criteria used across the reviews, leading to variation in the studies included (17 studies in the Tussing‐Humphreys review, 103 in this review).

There are several factors by which discrimination or differential health outcomes may occur that were either not captured at all or were only partially captured, in this review. Some factors, such as sexual orientation, were not measured in any of the 103 trials included in our review. This is despite there being known inequalities in weight by sexual orientation. For example, women who identify as lesbian are more likely to have overweight or obesity than women who identify as heterosexual.^151^, ^152^ The National Health Service in England has highlighted the need for further research to gain a better contextual understanding of weight issues in this group.^153^ Other factors that discrimination or differential health outcomes can occur by, such as gender and social capital, were only captured in a limited way. For example, gender was predominantly recorded in trials as either male or female, which does not reflect the full spectrum of gender. Similarly, despite its broad definition, social capital was only captured in trials as marital status, meaning that the full nature of people's personal support networks was not captured. Additionally, depending on the categorization of marital status, this measure may not reflect contemporary attitudes towards relationships and marriage. Similarly, our consideration of several PROGRESS‐Plus characteristics, such as place of residence (urban vs. rural), was binary, which loses detail in the complexity of people's circumstances and living arrangements. Future trials should consider broader categorizations of factors, such as of gender, to ensure demographic information fully represents how participants wish to identify.

Due to heterogeneity in intervention types and measures of the PROGRESS‐Plus criteria, such as country‐specific measures of SES or ethnicity, we deemed it was not appropriate to conduct a meta‐analysis. We suggest that future research should identify if a common range of measures covering the PROGRESS‐Plus criteria could be reported across trials of behavioral interventions, as well as identifying key stages of a trial (such as trial uptake, intervention adherence and outcome) for which differences in these measures should be reported. While reporting common measures across key stages of a trial will not overcome the issue that most trials may not have sufficient statistical power to identify if inequalities are present, more consistent reporting would facilitate future meta‐analyses that could address inequalities‐focused research questions.

### 4.1 Strengths and limitations

To our knowledge, this systematic review is the most up‐to‐date and comprehensive review investigating the association between indicators of inequality and behavioral weight management interventions published to date. In particular, it is the first to investigate the impact of inequalities at several stages of an intervention (such as trial or intervention uptake and follow‐up). Utilizing the PROGRESS‐Plus criteria ensured a comprehensive examination of inequality beyond the individual measures (such as SES^20^ or gender^16^) that previous reviews have focused on. In using “publication families,”^24^ we endeavored to capture all papers published from each included trial. Furthermore, we also contacted authors of trials included in this review to request any missing relevant data, aiding the completeness of our data collection.

Despite the usefulness of harvest plots in graphically synthesizing information across studies that cannot be meta‐analyzed, they are unable to overcome the limitation of low statistical power of the individual studies in detecting differential effects of interventions. This is pertinent when considering the impact inequalities have on trial/intervention uptake, adherence, attrition, and effectiveness, as studies are generally only sufficiently powered to detect a significant difference in weight outcomes between the intervention and control groups. This is likely attributable, in part, to analyses of inequalities rarely being part of the main analysis plan and such analyses often being performed post hoc. A large number of our included studies had a relatively small sample size (e.g., 41 studies had 0–200 participants). This may explain why a large number of studies included in our harvest plots found no inequality gradient for any of the outcomes we studied. It may be that an inequality gradient is present in some of these studies, but there was insufficient statistical power to detect it. Future research should consider alternative methods of data synthesis, such as individual participant data meta‐analysis, when addressing inequality‐focused research questions of intervention studies. In individual participant data meta‐analyses, different measures of an inequality criterion (such as SES) can be harmonized and pooled, providing greater statistical power to detect significant differences in uptake, adherence, attrition, and intervention effectiveness.

A further limitation of our review is that, although we took a comprehensive approach to considering various indicators of inequality and their interaction with behavioral weight management interventions, we did not consider weight status (e.g., higher BMI category vs. lower BMI category) as a factor where differential uptake, adherence, attrition, or effectiveness may occur. Higher weight status is associated with increased weight stigma, which is linked to worsened mental and physical health, and healthcare avoidance.^154^, ^155^, ^156^ Therefore, inequalities in behavioral weight management interventions may also exist in this group. Furthermore, we only set out to include studies from high‐income (OECD) countries, meaning that the results cannot be extrapolated to low‐ and middle‐income countries. Similarly, by using a minimum BMI cut‐off of 25 kg/m^2^, we may have excluded a number of studies conducted across Asia‐Pacific countries that typically use lower BMI cut‐offs for overweight (23–24.9 kg/m^2^) and obesity (≥25 kg/m^2^).^157^ Finally, we were unable to conduct any meaningful analysis of intersectionality—for which two or more characteristics where inequalities may occur interact and produce inequality that is distinct and specific from the inequalities arising from individual characteristics.^158^ Given RCTs are typically only sufficiently powered to detect an interaction between outcome and intervention arm, they are not designed in a way that facilitates consideration of intersectionality. It is, however, an important issue for future research to address intersectionality in terms of differential intervention outcomes, as well as building on recent research that explored intersectional differences in prevalence of obesity.^159^, ^160^, ^161^

## 5 CONCLUSION

We found that most trials of behavioral weight management interventions did not consider whether inequalities in trial or intervention uptake, adherence, trial attrition, or weight outcomes occurred by a measure of the PROGRESS‐Plus criteria. This is likely to have been because analyses of inequalities in trials are often post hoc and commonly are not included in the main analysis plan of a trial, as RCTs are generally only sufficiently powered to detect an interaction between trial arm and the primary outcome. In studies that did conduct such analyses, most found that no inequalities gradient was present. In the studies that did find a gradient, they mostly found that the intervention favored those who were “more advantaged” for uptake, adherence, and trial attrition. However, this was not the case for weight outcomes at 12‐month follow‐up, where there was a more equal balance between trials favoring more and less advantaged groups. These findings may suggest that behavioral weight management interventions are equitable for those who reach the 12‐month follow‐up. Future research should include standard measures of the PROGRESS‐Plus criteria and consider alternative methods of data synthesis, such as meta‐analysis of individual participant data, when addressing inequality‐focused questions in trials of interventions. This would help to overcome limitations such as insufficient statistical power, in order to detect potential differences by measures of inequalities.

## CONFLICT OF INTEREST

ALA is principal investigator on two publicly funded (NIHR, MRC) trials where the intervention is provided by WW (formerly Weight Watchers) at no cost. SJG is principal investigator on a publicly funded (NIHR) trial in which the intervention is provided by WW (formerly Weight Watchers) at no cost. MPK has undertaken consultancy for Slimming World, and led the obesity and weight management guidelines development for NICE from 2005 until 2014.

## Supporting information




**Table S1:** Detailed Study Characteristics
