# Supplementary material for: A systematic review of inequalities in the uptake of, adherence to, and effectiveness of behavioral weight management interventions in adults
Source: Obes Rev. 2022 Mar 3;23(6):e13438. doi: 10.1111/obr.13438 (PMC9285567; doi:10.1111/obr.13438)
Supplement: Supplementary file 1 — Table S1: Detailed Study Characteristics [file OBR-23-0-s001.docx]

A systematic review of inequalities in the uptake of, adherence to, and effectiveness of behavioural weight management interventions in adults

Jack M Birch^1^, Rebecca A Jones^1^, Julia Mueller^1^, Matthew D McDonald^2^, Rebecca Richards^1^, Michael P Kelly^3^, Simon J Griffin^1, 3^, Amy L Ahern^1^

^1^ MRC Epidemiology Unit, University of Cambridge, Cambridge, UK.

^2^ Curtin School of Population Health, Curtin University, Perth, Australia.

^3^ Primary Care Unit, University of Cambridge, Cambridge, UK.

Corresponding author:

Jack M Birch

MRC Epidemiology Unit, University of Cambridge School of Clinical Medicine, Box 285 Institute of Metabolic Science, Cambridge Biomedical Campus, Cambridge, CB2 0QQ

[jack.birch@mrc-epid.cam.ac.uk](mailto:jack.birch@mrc-epid.cam.ac.uk)

**Supplementary file 1a – Example search strategy (Ovid Medline)**

1 Obesity/

2 Obesity, Morbid/

3 Overweight/

4 Obesity, Metabolically Benign/

5 Weight loss/

6 obes$.ti.

7 overweight.ti.

8 weight.ti.

9 (adipos$ or body fat).ti.

10 (obes$ or overweight or weight loss).ti,ab.

11 limit 10 to ("in data review" or in process or "pubmed not medline")

12 1 or 2 or 3 or 4 or 5 or 6 or 7 or 8 or 9 or 11

13 Weight Reduction Programs/

14 Behavior Therapy/

15 Cognitive Therapy/

16 Counseling/

17 Directive Counseling/

18 Self-Help Groups/

19 counsel$.ti,ab.

20 (behav$ adj3 (therap$ or program$ or intervention$)).ti,ab.

21 Health Education/

22 Diet, Reducing/

23 Diet, Fat-Restricted/

24 Caloric Restriction/

25 Diet Therapy/

26 (diet$ adj counsel$).ti,ab.

27 (diet$ adj education$).ti,ab.

28 (nutrition$ adj counsel$).ti,ab.

29 (nutrition$ adj education$).ti,ab.

30 (nutrition$ adj intervention$).ti,ab.

31 (diet$ adj (modif$ or therapy or intervention$ or strateg$)).ti,ab.

32 ((diet or dieting or slim$) adj (club$ or organi?ation$)).ti,ab.

33 (weight reduc$ adj diet$).ti,ab.

34 (weightwatcher$ or weight watcher$).ti,ab.

35 Exercise/

36 Exercise Therapy/

37 Motor Activity/

38 Physical Conditioning, Human/

39 Physical Fitness/

40 physical activity.ti,ab.

41 (exercise adj3 (therap$ or program$ or intervention$)).ti,ab.

42 ((lifestyle or life style) adj (modification$ or intervention$)).ti,ab.

43 13 or 14 or 15 or 16 or 17 or 18 or 19 or 20 or 21 or 22 or 23 or 24 or 25 or 26 or 27 or 28 or

29 or 30 or 31 or 32 or 33 or 34 or 35 or 36 or 37 or 38 or 39 or 40 or 41 or 42

44 12 and 43

45 Obesity/dh, th, dt, rh [Diet Therapy, Therapy, Drug Therapy, Rehabilitation]

46 Obesity, Morbid/dh, th, dt, rh

47 Overweight/dh, th, dt, rh

48 (weight loss adj (intervention$ or program$ or trial$)).ti,ab.

49 (weight reduc$ adj (intervention$ or program$ or trial$)).ti,ab.

50 (weight management adj (intervention$ or program$ or trial$)).ti,ab.

**Supplementary file 1b – Data extraction form used**

**1. General Information**

| **Study ID:** | Date form completed: |
| --- | --- |
| Data extractor: | Data checker: |
| Study title: | |
| Other papers in publication family: | |
| Study design (delete as appropriate)                                                        Randomised Controlled Trial (RCT)  /  Cluster Randomised Controlled Trial (cluster RCT) | |
| Country of study: | |
| Funding source of study: | Potential conflict of interest from funding? Y / N / unclear |

| **Study Characteristics** | **Descriptions as reported in the paper(s)** | **Page/ Para/ Figure #** |
| --- | --- | --- |
| Weight Loss or Weight Loss Maintenance |  |  |
| Inclusion/Exclusion criteria for participants |  |  |
| Was the intervention targeted at a specific group? |  |  |
| Describe the intervention setting (e.g. commercial provider such as Weight Watchers, GP clinics, etc) |  |  |
| Study start and end dates |  |  |

**2. Intervention condition** (copy and paste for any additional groups)

| **Intervention characteristic** | **Descriptions as stated in the report/paper** | **Page/ Para/ Figure #** |
| --- | --- | --- |
| Intervention group name |  |  |
| Intervention overview |  |  |
| Group or individual delivery |  |  |
| Mode of delivery (e.g. web, face-to-face) |  |  |
| Timing (e.g. frequency, duration of each session and intervention) |  |  |
| Providers (e.g. profession and training received) |  |  |
| Co-interventions |  |  |
| Any further notes: |  |  |

**3. Control condition**

| **Control condition  characteristic** | **Descriptions as stated in the report/paper** | **Page/ Para/ Figure #** |
| --- | --- | --- |
| Intervention group name |  |  |
| Intervention overview |  |  |
| Group or individual delivery |  |  |
| Mode of delivery (e.g. web, face-to-face) |  |  |
| Duration of intervention |  |  |
| Timing (e.g. frequency, duration of each session) |  |  |
| Providers (e.g. profession and training received) |  |  |
| Co-interventions |  |  |
| Any further notes: |  |  |

**4. Study and Intervention Uptake**

**Uptake**

| **Participants**  Include if relevant | **Include information for each group (i.e. intervention and controls) under study** | **Page/ Para/ Figure #** |
| --- | --- | --- |
| - How many individuals agreed to participate? | Invited n =  Agree to participate n (%) = |  |
| - Total number randomised (or total pop. at start of study for NRCTs) |  |  |
| - Number allocated to each group | Intervention n (%) =  Control n (%) = |  |
| - For cluster RCTs, number of clusters, number of people per cluster | Intervention n (%) =  Control n (%) = |  |
| - Where there any significant baseline imbalances? | Yes/No/Unclear  Details: |  |
| - Number and reason for (and sociodemographic differences of) withdrawals and exclusions for each group | Intervention:  Control: |  |
| - Participants receiving the allocated intervention or exposure of interest (i.e. attending >1 session, actually downloading an app, etc) | Intervention arm n (%) = |  |
| - Did the study authors report differences by any of the PROGRESS-Plus factors? If yes, copy and paste results and say no differences, positive gradient or negative gradient |  |  |

**Attrition**

|  | **Description in paper** | **Page/ Para/ Figure #** |
| --- | --- | --- |
| Did the study authors report information on attrition in the study protocol? (if yes, state how they defined it, include if a dichotomous or continuous measure was used) |  |  |
| Group-level results for attrition (and by PROGRESS-Plus) |  |  |

**Adherence/Attendance**

|  | **Description in paper** | **Page/ Para/ Figure #** |
| --- | --- | --- |
| Did the study authors report information on attrition, adherence or attendance in the study protocol? (if yes, state how they defined it, include if a dichotomous or continuous measure was used) |  |  |
| Group-level results for adherence or attendance (and by PROGRESS-Plus) |  |  |

**Results**

Copy and paste the appropriate table for each outcome and subgroup at each timepoint, including baseline

**Dichotomous outcome (e.g. weight loss of five percent or greater)** page/para/fig

| Outcome (unit) |  | | | |  |
| --- | --- | --- | --- | --- | --- |
| Self-reported or Objective |  | | | |  |
| Timepoint |  | | | |  |
| Post-intervention or change from baseline? |  | | | |  |
| **Results** | **Intervention** | | **Comparison** | |  |
|  | Number achieving weight loss > 5% | No. participants | Number achieving weight loss > 5% | No. participants |  |
|  |  |  |  |  |  |
| P-value |  | | | |  |
| Did the study authors report differences by any of the PROGRESS-Plus factors? If yes, copy and paste results |  | | | |  |

**Continuous outcome (e.g. weight change in kg/ change in waist circumference)** page/para/fig (copy and paste if multiple continuous outcomes)

| Outcome |  | | | | | |  |
| --- | --- | --- | --- | --- | --- | --- | --- |
| Timepoint |  | | | | | |  |
| Self-reported or objective |  | | | | | |  |
| Post-intervention or change from baseline? |  | | | | | |  |
| **Results** | **Intervention** | | | **Comparison** | | |  |
|  | Mean (95% CI) | SD (or other variance) | No. participants | Mean (95% CI) | SD (or other variance) | No. participants |  |
|  |  |  |  |  |  |  |  |
| P-value |  | | | | | |  |
| Did the study authors report differences by any of the PROGRESS-Plus factors? If yes, copy and paste results |  | | | | | |  |

Details of further information required from author

|  |
| --- |

**Supplementary Table 1: Detailed Study Characteristics**

Key: *P – Place of residence, R – Race/ethnicity, O – Occupation, G – Gender/sex, Re – Religion, E – Education, SES – Socioeconomic status, SC – Social capital, + – plus other factors where discrimination may occur. WL – Weight loss; WLM – Weight loss maintenance*

| Author, year | Country | Sample size | Weight loss (WL) or weight loss maintenance (WLM) | Quality appraisal score | Group targeted | Variables reported at baseline | Differential measures reported for uptake | Differential measures reported for adherence and attendance | Differential measures reported for attrition variables | Differential measures reported for weight outcome |
| --- | --- | --- | --- | --- | --- | --- | --- | --- | --- | --- |
| Ackermann 2008 | USA | 92 | WL | Fair | - | R,G,+ | - | - | R,G,+ | R,G |
| Ackermann 2015 | USA | 509 | WL | Fair | Low-income | R,G,SES,  + | R,G,SES, + | R,G,SES, + | - | R |
| Ahern 2017 | UK | 1267 | WL | Fair | - | R,G,E,SES, + | R,G,E,SES, + | R,G,E,SES,+ | R,G,E,SES, + | G,E,SES |
| Anderson 2014 | UK | 329 | WL | Good | Aged 50 to 74 through colorectal screening programme | R,O,G,E, SES,SC,  + | - | - | - | O,G,SES, SC,+ |
| Appel 2011 | USA | 415 | WL | Good | - | R,O,G,E, SES,+ | - | - | - | - |
| Aveyard 2016 | UK | 1882 | WL | Fair | - | R,G,E,SES,  + | R,SES | SES | - | SES |
| Beeken 2017 | UK | 537 | WL | Fair | - | R,G,E,  SES,+ | - | - | G,SES, + | - |
| Bennett 2012 | USA | 365 | WL | Good | Socio-economically disadvantaged | R,O,G,E, SES,+ | G,+ | R,O,G,E, SES,  + (age, health literacy) | R,O,G,E,  SES,+ (age, language) | R,O,G,E, SES,+ (language) |
| Bhopal 2014 | UK | 171 | WL | Good | Individuals of South Asian descent | R,G,Re,E,  + | - | - | - | - |
| Burke 2005 | Australia | 241 | WL | Fair | - | G,+ | - | - | - | - |
| Cadmus-Bertram 2016 | USA | 105 | WL | Fair | Women with elevated breast cancer risk | R,G,E,SC,  + | - | - | - | - |
| Chirinos 2016 | USA | 230 | WL | Fair | Low-income | R,G,E,SES,  + | - | - | - | - |
| Christian 2011 | USA | 279 | WL | Fair | Health centres with largely Hispanic patients | R,G,+ | - | - | - | R,G,+ |
| Cohen 1991 | USA | 30 | WL | Fair | - | G,+ | - | - | - | G,+ |
| de Vos 2014 | Netherlands | 407 | WL | Fair | Women | R,G,E,SC,  + | - | - | - | - |
| Demark-Wahnefried | USA | 136 | WL | Good | Breast cancer survivors + daughters | R,G,E,SES,  + | R,+ | - | R,E,SES, + | - |
| Eaton 2016 | USA | 211 | WL | Fair | - | R,O,G,E,SES  + | - | - | - | - |
| Fischer 2016 | USA | 163 | WL | Fair | - | G,+ (age, language) | - | - | - | + (protocol language) |
| Fitzgibbon 2010 | USA | 213 | WL | Fair | Black women | R,O,G,E,SES,SC,+ | - | - | O,G,E,SES,SC,+ | - |
| Godino 2016 | USA | 404 | WL | Good | Young adults (18 to 35) | R,G,+ | - | - | - | - |
| Greaves 2015 | UK | 108 | WL | Fair | - | R,G,E,SES,  + | G,+ | - | - | - |
| Haapala 2009 | Finland | 125 | WL | Fair | - | G,E,SC,  + | - | - | - | - |
| Hunt 2014 | UK | 747 | WL | Good | Men | R,O,G,SES, SC,+ | - | - | - | R,O,E,SES, SC,+ |
| Huseinovic 2016 | Sweden | 110 | WL | Fair | Postpartum women | G,E,SC,  + | - | - | Education + | - |
| Jakicic 2011 | USA | 269 | WL | Fair | - | R,G,+ | - | - | - | - |
| Jansson 2012 | Sweden | 133 | WL | Fair | - | G,+ | - | - | - | - |
| Jebb 2011 | UK, Germany and Australia | 772 | WL | Fair | - | G,+ | - | - | + | - |
| Jeffery 1993 | USA | 202 | WL | Fair | - | R,E,SC,  + | - | G | G | G |
| Jenkins 2017 | Canada | 919 | WL | Fair | - | R,G,E,SES,  + | - | - | - | - |
| Jolly 2011 | UK | 740 | WL | Fair | - | R,G,SES,  + | - | - | R,G,SES, + | G |
| Jones 1999 | USA | 112 | WL | Fair | - | R,G,+ | - | - | - | - |
| Kanke 2015 | Japan | 50 | WL | Fair | - | G,E,+ | - | - | - | - |
| Katula 2011 | USA | 301 | WL | Good | - | R,G,E,  + | - | - | - | - |
| Knowler 2002 | USA | 2161 | WL | Good | - | R,O,G,SES,  SC,+ | - | - | - | R,G |
| Kuller 2012 | USA | 508 | WL | Good | Women | R,O,G,E,SC,  + | - | - | - | - |
| Kulzer 2009 | Germany | 182 | WL | Fair | - | G,E,+ | - | - | G,E,+ | - |
| Kumanyika 2012 | USA | 261 | WL | Fair | - | R,O,G,E,SC,  + | - | SES | - | - |
| Little 2016 | UK | 818 | WL | Fair | - | G,SES,  + | - | - | - | - |
| Logue 2005 | USA | 665 | WL | Fair | - | R,G,+ | - | - | - | - |
| Luley 2014 | Germany | 184 | WL | Fair | - | G,+ | - | - | - | - |
| Ma 2013 | USA | 241 | WL | Good | - | R,G,E,SES,  + | - | - | - | G |
| Marrero 2016 | USA | 225 | WL | Fair | - | R,G,E,SES,  SC,+ | - | - | - | - |
| Martin 2008 | USA | 144 | WL | Fair | Low income African American women | R,G,E,SC,  + | - | - | E,SC, + | - |
| Mensink 2003 | Netherlands | 114 | WL | Fair | - | G,+ | G,+ | - | - | - |
| Mitsui 2008 | Japan | 46 | WL | Fair | - | G,+ | - | - | - | - |
| Moore 2003 | UK | 843 | WL | Fair | - | G,SES,  + | - | - | - | - |
| Morgan 2011 | Australia | 65 | WL | Fair | Men | O,G,SES,  + | - | O,+ | + | - |
| Nakade 2012 | Japan | 235 | WL | Fair | - | G,+ | - | - | - | G |
| Nanchahal 2012 | UK | 381 | WL | Fair | - | R,O,G,E,SES,SC,+ | - | - | R,O,G,E, SES,SC, + | - |
| Nicklas 2014 | USA | 75 | WL | Fair | - | R,G,E,SES,  SC,+ | - | - | - | - |
| Nilsen 2011 | Norway | 213 | WL | Fair | Women with recent gestational diabetes mellitus | O,G,E,SC,  + | - | - | - | - |
| O'Brien 2017 | USA | 63 | WL | Good | Hispanic women, majority foreign born and low income | R,G,E,SES,  + (age, country of origin) | - | - | - | - |
| Ockene 2012 | USA | 312 | WL | Fair | Latino community | R,O,G,E,SC,  + | - | - | G | - |
| Pacanowski 2015 | USA | 162 | WL | Fair | - | R,G,E,  + | - | - | - | O |
| Parikh 2010 | USA | 99 | WL | Fair | Low-income, high ethnic minority community | R,O,G,SES,  + (age, language, food sufficiency) | - | - | G,+ | - |
| Patrick 2011 | USA | 441 | WL | Fair | Men | R,G,E,SC,  + | R,SC, + | - | R,+ | - |
| Penn 2009 | UK | 102 | WL | Fair | - | O,G,SES,  + | - | - | + | - |
| Phelan 2017 | USA | 370 | WL | Good | Low-income postpartum women | R,O,G,SES,  SC,+ | R,+ (language) | R | R,O,SES,SC,+ | R,O,SES,SC, + |
| Puhkala 2015 | Finland | 113 | WL | Fair | Male truck and bus drivers | G,+ | - | - | - | - |
| Rock 2007 | USA | 70 | WL | Fair | Women | R,G,E,SC,  + | - | - | - | - |
| Rock 2015 | USA | 697 | WL | Good | Women who were breast cancer survivors | R,G,+ | - | - | - | - |
| Rodriguez-Cristobal 2017 | Spain | 864 | WL | Fair | - | G,+ | - | - | - | - |
| Rosas 2015 | USA | 207 | WL | Good | Low-income Latinos | R,O,G,E,SES,+ (age, food security, birthplace) | - | - | G | G |
| Ross 2012 | Canada | 490 | WL | Fair | - | G,+ | - | G | G,+ | G |
| Shapiro 2012 | USA | 170 | WL | Fair | - | R,G,E,  + | - | G,E, + | G,E,+ | - |
| Silva 2010 | Portugal | 239 | WL | Fair | Women | G,E,SC,  + | - | - | E,SC, + | - |
| Stevens 1993 | USA | 564 | WL | Good | - | R,O,G,SC,  + | - | G | - | R,G |
| Stevens 2001 | USA | 1191 | WL | Good | - | R,G,E,  + | R,O,G,E, SC,+ | - | - | R,G,+ |
| Svetkey 2015 | USA | 365 | WL | Good | Young adults (18 to 35) | R,O,G,E,SES,SC,+ | - | - | - | R,G,E,SES, + |
| Thomas 2017 | USA | 271 | WL | Good | - | R,G,E,  + | - | - | R,G,E, + | - |
| Tsai 2010 | USA | 50 | WL | Good | - | R,E,+ | - | - | - | - |
| Tuomilehto 2001 | Finland | 522 | WL | Good | - | G,+ | - | - | - | - |
| van Wier 2011 | Netherlands | 1386 | WL | Fair | "Dutch service-sector employees" | G,E,SC,  + (age, birthplace) | - | - | - | - |
| Venkat Narayan 1998 | USA | 95 | WL | Fair | Pima Indians | R,O,G,  + | G | O | - | - |
| von Gruenigen 2012 | USA | 75 | WL | Fair | Endometrial cancer survivors (women) | R,O,G,E,SES,SC,+ | - | - | - | - |
| Wadden 2011 | USA | 261 | WL | Good | - | R,G,E,  + | - | - | - | - |
| Whelton 1998 | USA | 585 | WL | Good | Older persons (aged 60 to 80) | R,G,E,  + | - | - | - | R,G |
| Wing 1998 | USA | 154 | WL | Fair | - | G  + | - | - | - | - |
| Wylie-Rosett 2001 | USA | 588 | WL | Fair | - | R,G,E,  + | R,G,E,  + | - | R,G,E,SC, + | R,G |
| Yeh 2016 | USA | 60 | WL | Fair | Chinese immigrants | R,G,  + | - | - | - | - |
| Cussler 2008 | USA | 135 | WLM | Fair | Middle-aged women | G,+ | - | - | - | - |
| Pekkarinen 2015 | Finland | 201 | WLM | Fair | - | O,G,E,SC,  + | - | - | - | O,G,E, + |
| Perri 1988 | USA | 123 | WLM | Fair | - | G,+ | - | - | - | - |
| Sherwood 2013 | USA | 419 | WLM | Good | - | G,+ | - | - | - | - |
| Simpson 2015 | UK | 166 | WLM | Fair | - | R,G,+ | - | - | - | - |
| Svetkey 2008 | USA | 1032 | WLM | Good | - | R,G,E,SES,  + | - | R,G,E,  SES, + | R,G,E,SES, + | R,G,+ |
| Voils 2017 | USA | 222 | WLM | Fair | - | R,O,G,E,SC,  + | - | - | - | - |
| Wing 2006 | USA | 314 | WLM | Fair | - | G,+ | - | - | - | - |
| Young 2017 | Australia | 92 | WLM | Good | - | O,G,E,SES,  SC,+ (age, birthplace, language) | - | - | O,G,E,SES,SC,+ (age, place of birth, language at home) | SES,+ |
| Astbury 2018 | UK | 278 | WL | Fair | - | R,G,SES,  + | G,SES, + | G,SES, + | G,SES, + | G,SES, + |
| Bennett 2018 | USA | 351 | WL | Fair | - | R,O,G,E,SES,SC,+ | - | - | - | R,G |
| Daley 2019 | UK | 583 | WLM | Fair | - | R,O,G,SES, SC,+ | - | - | R,G,E,SES, SC | G |
| Fernandez-Ruiz 2018 | Spain | 74 | WL | Good | - | O,G,E,SC,  + | - | - | - | - |
| Fjeldsoe 2019 | Australia | 228 | WL | Fair | - | P,R,O,G,E, SES,+ (age, language) | - | - | P,R,O,G,E, SES,+ (age, language at home) | - |
| Godino 2019 | USA | 298 | WL | Good | - | R,O,G,E,SES,SC,+ (age, language) | - | - | - | R,G,+ (age, language at home) |
| Haire-Joshu 2018 | USA | 230 | WL | Fair | Mothers of preschool-aged children | R,G,E,SES, SC,+ | - | - | - | R,SES |
| Mai 2018 | Germany | 143 | WLM | Fair | - | G,+ | - | - | - | G,+ |
| Nakata 2019 | Japan | 119 | WLM | Fair | - | G,+ | - | - | - | - |
| Sniehotta 2019 | UK | 288 | WLM | Good | - | O,G,E,SES,  + | - | - | - | - |
| Tapsell 2017 | Australia | 251 | WL | Fair | - | R,G,E,  + (age, birthplace) | - | - | - | G |
| Tarraga Marcos 2017 | Spain | 180 | WL | Fair | - | G,+ | - | - | - | - |
| Teeriniemi 2018 | Finland | 532 | WL | Fair | - | R,G,+ | - | - | - | - |
| Viglione 2019 | USA | 45 | WL | Fair | Veterans | R,O,G,SES,  + (age, food security) | - | R,G,SES, + (age, food security) | - | - |

*Table 1 PROGRESS-Plus measures reported in included studies*

*P – Place of residence, R – Race/ethnicity, O – Occupation, G – Gender/sex, Re – Religion, E – Education, SES – Socioeconomic status, SC – Social capital, + – plus other factors where discrimination may occur. WL – Weight loss; WLM – Weight loss maintenance*
